# Identification of LBX2 as a novel causal gene of lung adenocarcinoma

**DOI:** 10.1111/1759-7714.13506

**Published:** 2020-06-22

**Authors:** Jingwen Hu, Yongkang Bai, Quanli Zhang, Ming Li, Rong Yin, Lin Xu

**Affiliations:** ^1^ Department of Thoracic Surgery The Affiliated Cancer Hospital of Nanjing Medical University, Jiangsu Cancer Hospital, Cancer Institute of Jiangsu Province Nanjing China; ^2^ Department of Scientific Research The Affiliated Cancer Hospital of Nanjing Medical University, Jiangsu Cancer Hospital, Cancer Institute of Jiangsu Province Nanjing China

**Keywords:** The Cancer Genome Atlas, epithelial to mesenchymal transitionLBX2lung adenocarcinoma

## Abstract

**Background:**

Lung adenocarcinoma (LUAD) is the most predominant histological type of lung cancer with a poor prognosis. In this study, we demonstrate that LBX2 regulates cell proliferation, migration and invasion and the potential molecular mechanism in LUAD.

**Methods:**

The Cancer Genome Atlas dataset was accessed to screen for novel genes and immunohistochemistry (IHC) assays were performed to determine the association between LBX2 expression and clinicopathological features of LUAD. 5‐ethynyl‐2′‐deoxyuridine, colony formation and Real Time xCELLigence analysis system were used to evaluate the cell proliferation abilities of LUAD. Wound healing, transwell and Matrigel assays were used to detect cell migration and invasion capacities. Xenograft tumor models were used to assess the oncogenic role of LBX2 in vivo.

**Results:**

We found that LBX2 was hyperexpressed in LUAD and correlated with clinicopathological features and poor prognosis in LUAD patients. Knockdown of LBX2 inhibited cell proliferation, migration and invasion of LUAD, whereas ectopic expression of LBX2 enhanced tumor growth, migration, and invasion. We further found that LBX2 might participate in epithelial‐to‐mesenchymal transition (EMT) progression and influence EMT‐related gene expression.

**Conclusions:**

The current study suggests that LBX2 plays an oncogenic role in LUAD and may participate in tumor proliferation, migration, and invasion through EMT progression.

**Key points:**

**Significant findings of the study**

LBX2 might participate in LUAD cell proliferation, migration and invasion via EMT progression.

**What this study adds**

LBX2 may represent a potential biomarker and a promising therapeutic target for LUAD.

## Introduction

Lung cancer is very commonly diagnosed and is the leading cause of cancer‐related death worldwide.^1^, ^2^, ^3^ The five‐year survival rate is less than 15%, even when patients have undergone standardized treatments.^4^ Lung adenocarcinoma (LUAD) is the most common histological type among lung cancers and accounts for approximately 40% of lung cancer cases.^5^ In recent years, several molecular targeting agents and immune checkpoint inhibitors (ICIs) have been approved and widely used to treat stage III–IV LUAD patients. However, the therapeutic effects can only extend the survival time by a few months and the overall survival (OS) remains poor.^6^, ^7^ Thus, further exploration of the molecular mechanisms underlying LUAD malignant progression, together with a better understanding of the alterations leading to this type of cancer, are crucial for the development of diagnostic biomarkers and therapeutic targets for LUAD patients.

To date, several LUAD‐associated genes have been described or documented. Most of these genes are involved in the regulatory networks needed for tumorigenesis, proliferation and migration, angiogenesis, and other tumor developmental processes.^8^, ^9^, ^10^, ^11^ To screen for novel genes related to LUAD, we comprehensively analyzed The Cancer Genome Atlas (TCGA) and found that ladybird‐like homeobox gene 2 (LBX2) might be a potential oncogene in LUAD. The LBX2 protein belongs to a homeodomain‐containing family of transcription factors known to play a crucial role in various biological processes. For example, LBX2 has been suggested to play a key role in the regulation of hypaxial myogenic precursor cell migration and muscle cell differentiation in zebrafish.^12^ Moreover, LBX2 deficiency may cause an abnormal development of the heart through influencing the migration of neural crest cells and affecting the process of cardiac septation.^13^ However, to date, limited information is available regarding the oncogenic role of LBX2. In this study, we isolated the LBX2 gene and analyzed its expression and potential pathogenic function throughout LUAD development. We identified LBX2 as a novel regulator of malignant properties in LUAD. Both in vitro and in vivo tumorigenic assays revealed that LBX2 could promote cell growth and migration and invasion by modulating epithelial to mesenchymal transition (EMT) progression. Collectively, these data demonstrated that LBX2 could be used as a biomarker and therapeutic target for combating LUAD in the future.

## Methods

### Data source and bioinformatics analysis

A TCGA dataset named TCGA_LUAD_exp_HiSeqV2‐2015‐02–24 was downloaded from the UCSC Cancer Browser (http://genomecancer.ucsc.edu/). This dataset contains information from 511 LUAD samples and 58 adjacent normal samples. The normalized gene expression of LBX2 was obtained from a “GenomicMatrix” file. Additionally, a Gene Expression Omnibus (GEO) microarray dataset (GSE21933) was obtained from the NCBI GEO website (https://www.ncbi.nlm.nih.gov/geo/) and the GSEA software was used to analyze the microarray data according to the developer's instructions. The C2tft (transcript factor target) calculation was applied and the highest factor was normalized.

### Tissue samples and immunohistochemistry assays

A total of 92 paired LUAD tissues and adjacent normal tissues were collected from patients who had undergone lobectomy and segmentectomy in the Department of Thoracic Surgery of the Cancer Hospital Affiliated to Nanjing Medical University, from January 2013 to October 2014. All tissues were diagnosed by two experienced pathologists, and the clinicopathological data of the patients were collected from their medical records system.

This study was approved by The Ethics Committee of Nanjing Medical University. Written informed consents were obtained from all patients.

For immunohistochemistry (IHC) assays, tissue sections were deparaffinized and rehydrated by a series of graded alcohol solutions. Endogenous peroxidase activity was blocked by incubating in 3% H_2_O_2_ and antigen retrieval was implemented by 0.01 M citrate buffer (pH 6.0). Primary polyclonal anti‐LBX2 (Invitrogen Bio; California, US, Cat: PA5‐60662, 1:50) was administered to search for positive cells. Two experienced pathologists independently scored the IHC results. As previously described, staining intensity was scored 0 (negative staining), 1 (weak staining), 2 (moderate staining), or 3 (high staining). The final score was obtained from the product of the percentage of positive cells and their staining intensity. Results ranged from 0–300.

### RNA extraction and qRT‐PCR

Total RNA was extracted from cultured cells and tissue samples using TRIzol reagent (Invitrogen) according to the manufacturer's instructions. RNA was reverse transcribed to cDNA using a Reverse Transcription Kit (Takara Bio; Kyoto, Japan). qRT‐PCR analyses were performed by using the SYBR Select Master Mix (Applied Bio; Massachusetts, US) and different sets of primers (listed in Table S1). The relative mRNA expression was normalized to that of β‐actin through the 2^‐ΔΔCT^ method. qRT‐PCR assays were performed in triplicate for each sample.

### Cell cultures

LUAD cell lines (A549, NCI‐H1299, SPC‐A1 and PC9) and the human bronchial epithelial (HBE) cell line were purchased from the American Type Culture Collection (ATCC). A549, NCI‐H1299 and PC9 cells were cultured in RPMI 1640 medium (HyClone Bio; Utah, US), whereas SPC‐A1 and HBE cells were cultured in DMEM medium (HyClone). Both media were supplemented with 10% fetal bovine serum (FBS; Gibco Bio; New York, US). All cells were cultured in a humidified incubator at 37°C with 5% CO_2_.

### siRNA, plasmid transfection and stable transfection technique

siRNA molecules targeting LBX2 were synthesized and purchased from RiboBio (Guangzhou, China). The synthesized siRNAs and negative control (NC) siRNA were transfected into LUAD cells using the Lipofectamine RNAiMAX transfection reagent (Invitrogen) according to the manufacturer's instructions. The pENTER plasmid with the full length human LBX2 cDNA (Realgene Bio; Shanghai, China) was used to overexpress LBX2. Plasmid vectors were extracted using DNA Midiprep kits (E.Z.N.A Endo‐Free Plasmid Mini Kit II, OMEGA Bio; Connecticut, US) and transfected using Lipofectamine 3000 (Invitrogen), following the manufacturer's protocol. All siRNA sequences used are listed in Table S2. Recombinant lentivirus were used for stable transfection of LBX2 were conducted and purchased from GenePharma (Shanghai, China).

### Western blotting

Cells were harvested and lysed in ice with a lysis buffer (RIPA, KeyGEN; Nanjing, China) containing 1 mM phosphatase inhibitor (PMSF, KeyGEN). The protein concentration of each sample was determined using a bicinchoninic acid kit (BCA, KeyGEN). Equal amounts of protein samples containing 20–40 μg of lysate protein were separated by 10% SDS‐PAGE gels and transferred onto polyvinylidene difluoride membranes (Pall Cor; New York, US). Membranes were blotted overnight at 4°C with the following primary antibodies: anti‐β‐actin (Cell Signaling Technology, Massachusetts, US Cat: 3700; 1:1000), anti‐LBX2 (Invitrogen, Cat: PA5‐69480; 1:1000), anti‐E‐cadherin (Cell Signaling Technology, Cat: 14472; 1:1000), anti‐N‐cadherin (Cell Signaling Technology, Cat: 13116s; 1:1000), and anti‐vimentin (Cell Signaling Technology, Cat: 5741; 1:1000).

### 5‐ethynyl‐2′‐deoxyuridine (EDU) assay

Transfected cells were placed in 96 well plates (8000 cells per well) and incubated with a 50 μM EDU solution for two hours. Next, 4% paraformaldehyde was used to fix the cells and 2 mg/mL glycine was added to neutralize the solution. Later, the cells were washed three times with 0.5% Triton X‐100 and incubated with 1 × Apollo reaction solution and 1 × Hoechst 33342 solution (Abcam Bio; London, UK). Finally, PBS was used to wash the cells thrice and a fluorescence microscope was used for imaging and calculating the proliferation rate of the cells.

### Colony formation assay

A total of 200 transfected cells were seeded in a six‐well plate and maintained in 100 μL of a medium containing 10% FBS. This medium was replaced every five days. After 10–14 days, cells were fixed with 4% paraformaldehyde and stained using a 0.1% crystal violet solution. Visible stained colonies were counted and used to evaluate cell proliferation.

### Real time xCELLigence analysis system

The real Time xCELLigence analysis system (RTCA) was used to evaluate cell proliferation. Transfected cells were seeded in E‐Plate 16 plates (8000 cells per well) and 100 μL medium containing 10% FBS was added to each well. Plates were locked into an RTCA DP device and an RTCA analyzer recorded the resulting “cell index” values.

### Wound healing assay

Transfected cells (40 000 cells) were plated in six‐well plates and scratched to induce wounds with a sterile 200 μL pipette tip. Images of wounds were captured at 0 and 24 hours after scratching. The coverage of the spacing of the gaps was measured at three different positions. All assays were repeated in triplicates.

### Cell migration and invasion assays

For migration assays, 24‐well plates were placed below Boyden chambers containing transwell membrane filters (Millipore Bio; Massachusetts, US). For invasion assays, a diluted Matrigel solution (BD Bio; New Jersey, US) was used. Approximately 40 000 transfected cells were plated on 8 mm pore size top chambers in 200 μL serum‐free, EGF‐free RPMI 1640 medium. The bottom chamber was filled with 800 μL RPMI 1640 medium containing 10% FBS. Migration and invasion were assayed, and the cells were stained after 48 hours.

### Animal studies

All animal experiments were conducted in a specific pathogen‐free (SPF) Laboratory Animal Center at Nanjing Medical University and approved by the Animal Care and Ethics Committee of Nanjing Medical University. All animals were manipulated in accordance with the guidelines of the U.S. National Institutes of Health. Transfected cells were harvested and 2 × 10^6^ cells were injected subcutaneously into each mouse used (six‐to eight‐week‐old mice). Tumors were collected six weeks after injection. Both the weight and volume of the subcutaneously‐induced tumors were measured, and the number of organic tumors formed was counted.

### Statistical analysis

Data are presented as the mean ± S.D. A two‐tailed Student's *t*‐test was used to determine statistical significance between two groups. A chi‐square test was performed to analyze the categorical data. The Kaplan–Meier analysis, log‐rank test, and Cox regression analysis were used to evaluate the OS; *P* < 0.05 was considered statistically significant. Data graphs were constructed using GraphPad Prism v.5.0 software.

## Results

### LBX2 is overexpressed in LUAD tissues and associated with poor prognosis in LUAD patients

**Figure 1:**
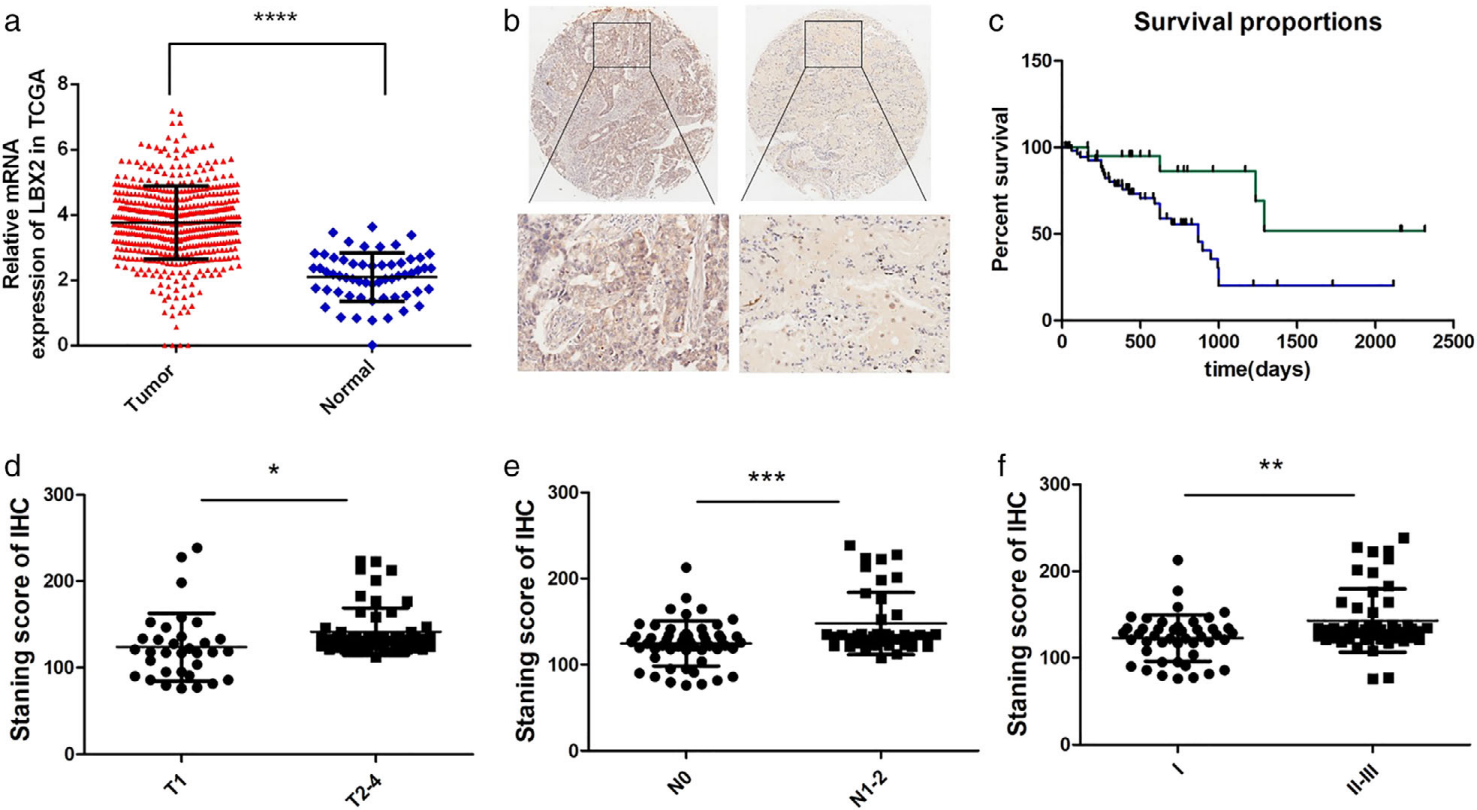
LBX2 is overexpressed in lung adenocarcinoma and correlates with clinicopathological characteristics. (**a**) LBX2 was hyperexpressed in LUAD tissues compared with adjacent normal tissues in the TCGA database (*P* < 0.0001). (**b**) Overexpression of LBX2 in LUAD tissues were detected by immunohistochemistry (IHC). (**c**) Lung adenocarcinoma patients with low expression of LBX2 have a higher percentage of overall survival compared with high LBX2 expression patients (*P* = 0.0075) (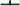) LBX2‐low, (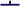) LBX2‐high. (**d**) Compared with T1 tumors, LBX2 was significantly upregulated in T2, T3, and T4 tumors (*P* = 0.0136). (**e**) N1–2 tumors had higher expression of LBX2 than those with N0 tumors (*P* = 0.005) (**f**) LBX2 showed higher expression in tumors at a more advanced TNM stage (*P* = 0.030).

By analyzing the TCGA_LUNG_exp_HiSeqV2‐2015‐02–24 dataset, we found that compared with that in normal adjacent tissues (*n* = 58), the mean expression level of LBX2 in LUAD tissues (*n* = 511) was significantly upregulated (*P* < 0.0001, Fig 1(a)). To explore the clinicopathological value of LBX2 in LUAD, we collected 92 paired LUAD and normal tissues from the Jiangsu Cancer Hospital. IHC assays revealed that the LBX2 protein levels were increased in LUAD tissues compared with those in normal adjacent tissues (in 87/92 paired tissues) (Fig 1b). IHC final LBX2 expression scores in LUAD tissues were defined as with “high LBX2 expression” when the final score was >120 and with “low LBX2 expression” when the final score was ≤120. We observed that the OS of patients with high LBX2 protein expression was worse than that of patients with low LBX2 protein expression (Fig 1c). Multivariate analyses revealed that the LBX2 expression level was related to T stage (*P* = 0.0136), N stage (*P* = 0.005), and TNM stage (*P* = 0.030) (Fig 1d–f). These results showed that overexpression of LBX2 predicts poor prognosis and could serve as an independent molecular marker for determining the OS of LUAD patients.

### LBX2 is highly expressed in LUAD cell lines

**Figure 2:**
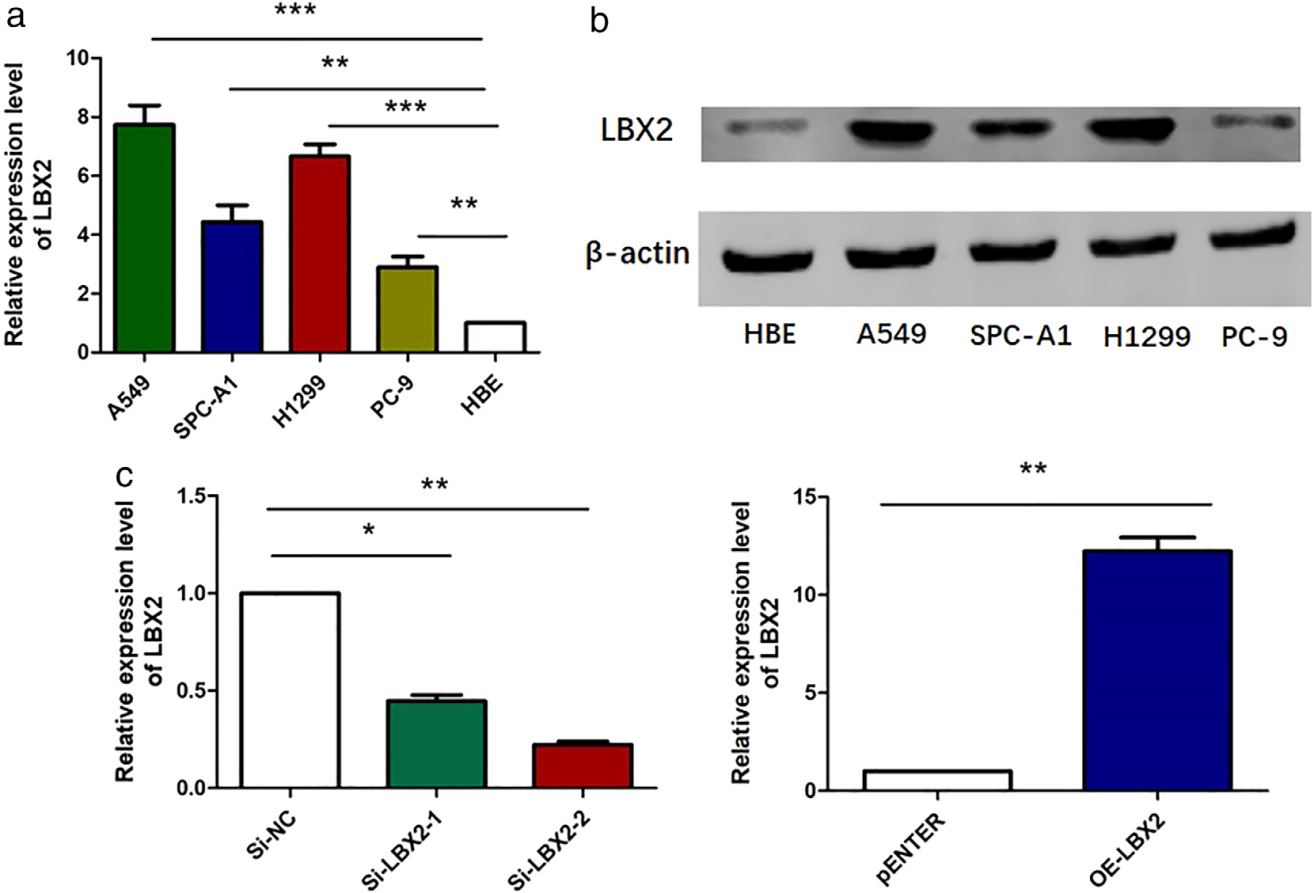
LBX2 is hyperexpressed in LUAD cell lines. (**a**) LBX2 mRNA were upregulated in A549, SPC‐A1, H1299, PC9 cell lines. (**b**) LBX2 protein expression level was hyperexpressed in LUAD cell lines. (**c**) Two specific siRNA both effectively downregulated the expression of LBX2 in mRNA level and the pENTER vector containing total coding sequence of LBX2 increased the mRNA level of LBX2.

We tested the relative expression of both LBX2 mRNA and the LBX2 protein in A549, SPC‐A1, NCl‐H1299, and PC9 LUAD cell lines and in HBE cells. The results showed that compared with HBE cells, the expression levels of LBX2 mRNA and LBX2 were significantly increased in all four LUAD cell lines (Fig 2a,b). Then, we designed two siRNAs (Si‐LBX2‐1 and Si‐LBX2‐2) and a pENTER plasmid (OE‐LBX2) to either knockdown or overexpress LBX2 in cells, respectively. qRT‐PCR assays were used to evaluate the efficiency of transfection (Fig 2c).

### LBX2 promotes tumor proliferation in LUAD cells in vitro

**Figure 3:**
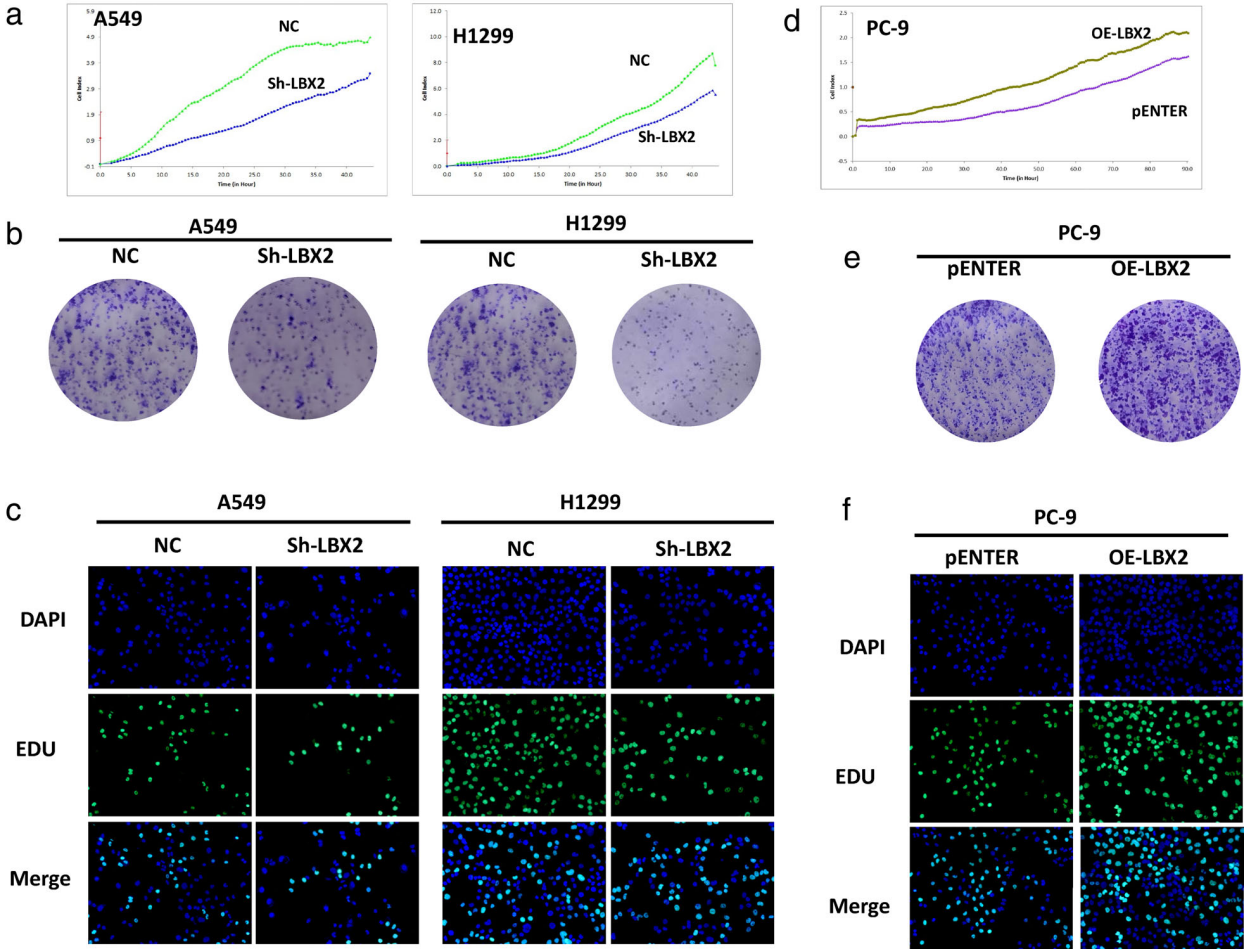
Knockdown of LBX2 suppresses proliferation of lung adenocarcinoma in vitro, while ectopic expression of LBX2 shows the reverse effect. (**a**) Real‐time cellular analysis (RTCA) revealed that knockdown of LBX2 inhibited proliferation of LUAD cells. (**b**) In colony formation assay, colony numbers of sh‐LBX2 group were less than the control group. (**c**) A549 and H1299 cells transfected with shRNA showed a lower proportion of EdU. (**d**) RTCA revealed that overexpression of LBX2 promoted proliferation of LUAD cells. (**e**) Colony numbers of the OE‐LBX2 group were higher than the control group. (**f**) PC‐9 cells transfected with pENTER vector showed a higher proportion of EdU.

We performed some experiments to explore the function of LBX2 in LUAD. We observed that knocking down LBX2 could inhibit cell proliferation in A549 and NCl‐H1299 cell lines; this result was validated by an RTCA system (Fig 3a), a colony formation assay (Fig 3b), and the EDU assay (Fig 3c). Moreover, ectopic expression of LBX2 promoted LUAD cell proliferation in the PC‐9 cell line (Fig 3d–f).

### LBX2 promotes tumor migration and invasion progression in LUAD cells in vitro

**Figure 4:**
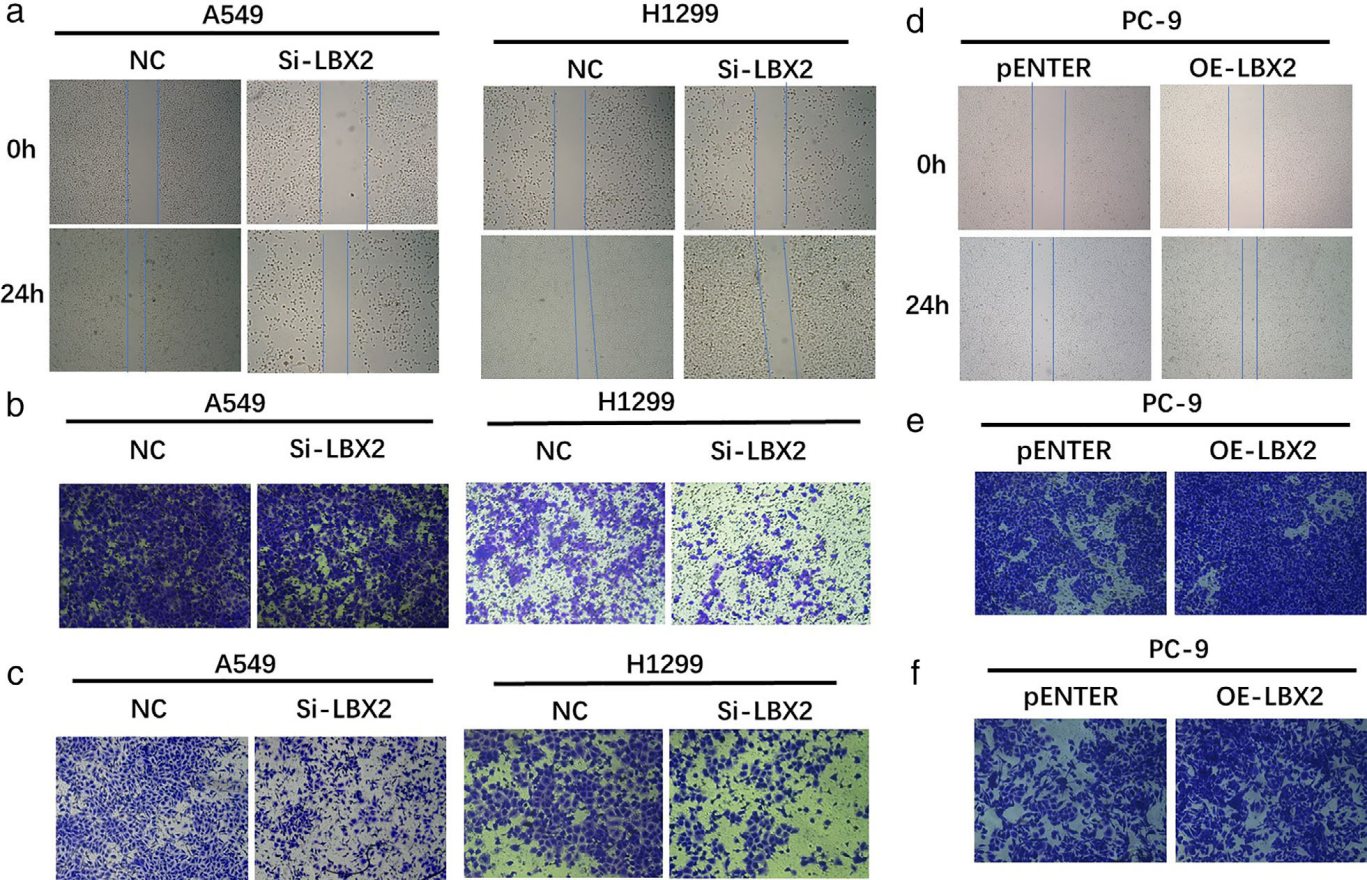
LBX2 promotes cell migration and invasion of lung adenocarcinoma in vitro. (**a**) The wound healing assay showed that knockdown of LBX2 inhibited the migration abilities of LUAD cells. (**b**). In the transwell assay, knockdown of LBX2 decreased the migratory rate of LUAD cells. (**c**) Matrigel assay suggested that LUAD cells transfected with siRNA showed a lower invasion rate. (**d**) The wound healing assay showed that overexpression of LBX2 promoted the migration abilities of LUAD cells. (**e**) In the transwell assay, ectopic expression of LBX2 was able to increase the migratory rate of LUAD cells. (**f**) Matrigel assay suggested that LUAD cells transfected with pENTER vector showed a higher proportion rate of invasion (*P < 0.05; **P < 0.01).

We also found that knocking down LBX2 could inhibit cell migration in A549 and NCl‐H1299 cell lines, as determined by wound healing (Fig 4a) and transwell assays (Fig 4b). Then, we performed Matrigel assays to determine cell invasion abilities. The results showed that knocking down LBX2 could inhibit LUAD cell invasion (Fig 4c). Moreover, overexpression of LBX2 could promote cell migration and invasion in PC‐9 cell line (Fig 4d–f). These data suggest that LBX2 plays a critical role in proliferation, migration, and invasion of LUAD cells.

### LBX2 promotes LUAD tumor growth in vivo

**Figure 5:**
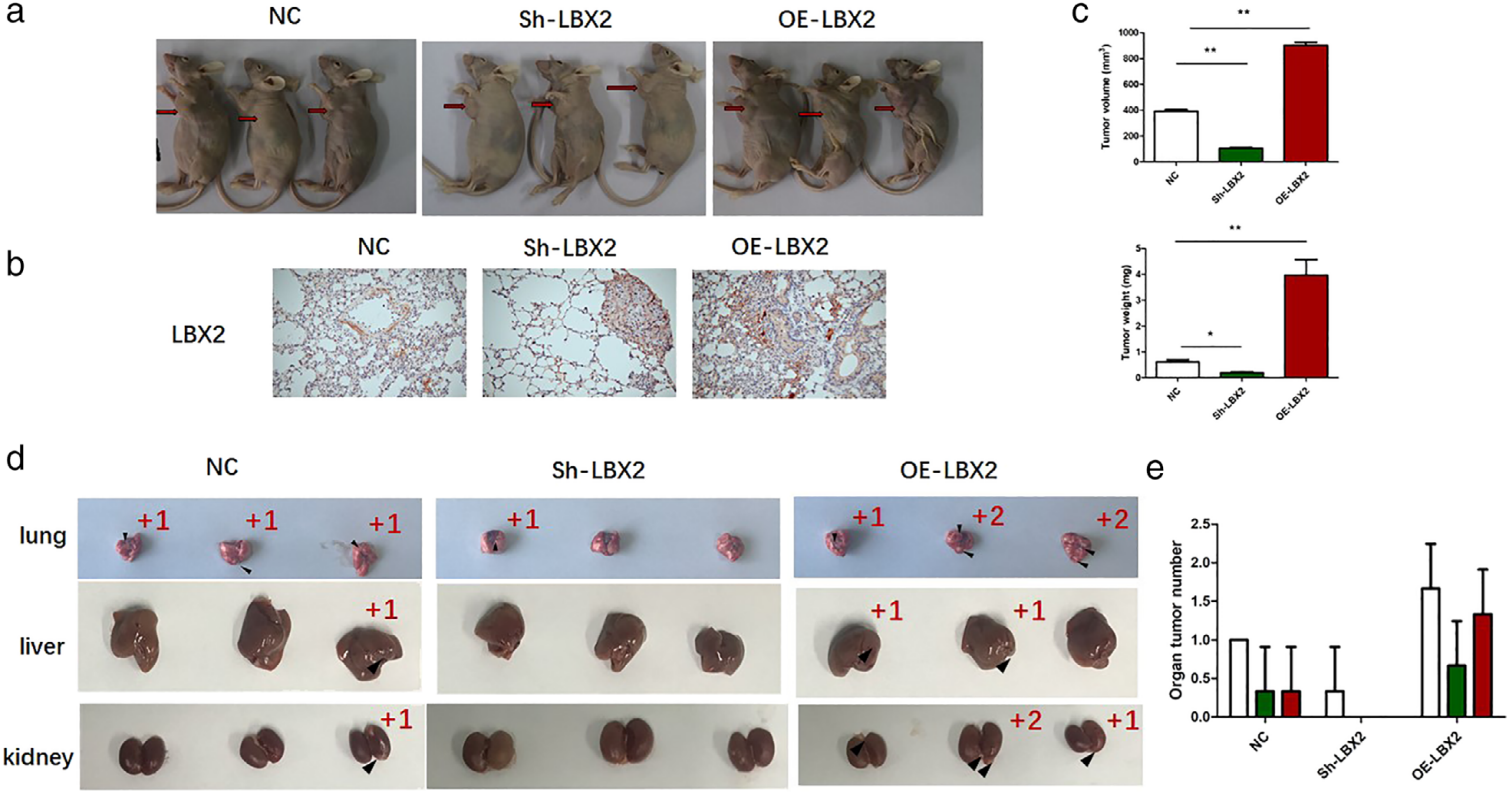
LBX2 promotes proliferation, migration and invasion of lung adenocarcinoma in vivo. (**a**) Xenograft tumors obtained from sh‐LBX2 transfected A549 cell‐treated mice had smaller tumors than the control group and tumors from OE‐LBX2 had larger tumors than the control group. (**b**). IHC of tumor nodules shown LBX2 was downregulated in sh‐LBX2 cells and upregulated in OE‐LBX2 cells. (**c**) The volume and weight of tumor nodules of each group were measured and the results revealed that LBX2 promoted proliferation of LUAD in vivo. (**d,e**). Knockdown of LBX2 decreased metastatic activity to lung, liver and kidney tissues from subcutaneous xenografts, whereas overexpression of LBX2 greatly enhanced metastatic activity to lung, liver and kidney tissues (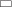) lung, (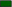) liver, (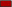) kidney.

Xenograft tumor models were used to assess the oncogenic role of LBX2 in vivo. A549 cells transfected with stable plasmids (OE‐LBX2) or shRNAs (Sh‐LBX2) were injected subcutaneously into nude mice (Fig 5a). After six weeks, mice were dissected and their subcutaneous and organic tumors were harvested. LBX2 protein levels in the tumor xenografts were verified by IHC (Fig 5b). We found that LBX2 silencing could inhibit subcutaneous tumor growth (volume and weight) (Fig 5c) and reduce visible organic metastatic lesions (in quantity) (Fig 5d,e) in vivo. Moreover, overexpression of LBX2 evidently promoted tumor proliferation and metastatic activity in vivo (Fig 5a–e). Taken together, these data indicate that LBX2 promotes LUAD tumor growth and metastatic activity in vivo.

### LBX2 might exert its oncogenic role in LUAD via EMT progression

**Figure 6:**
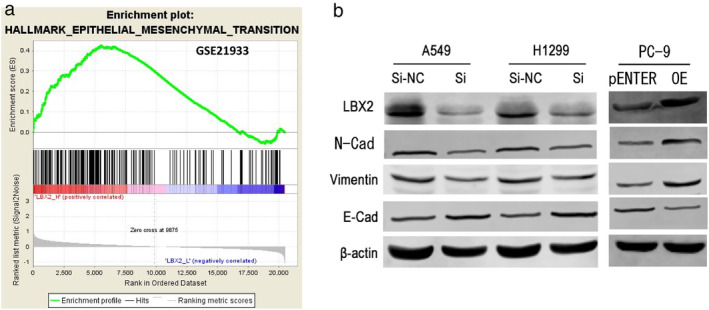
LBX2 might regulate EMT progression, leading to increased cell proliferation, migration and invasion of LUAD. (**a**) GSEA analysis revealed that LBX2 expression was highly correlated with EMT process (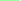) Enrichment profile, (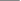) Hits, (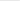) Ranking metric scores. (**b**) Western blot showed that knockdown of LBX2 increased the expression of E‐cadherin and decreased the expression of N‐cadherin and vimentin, and ectopic expression of LBX2 showed the reverse effect.

As the function of the LBX2 gene is unknown, we used GSEA to analyze microarray data (GEO microarray dataset GSE21933). The GSEA output indicated that the overexpression of LBX2 was positively correlated with EMT progression in LUAD (Fig 6a). Furthermore, EMT was proven to be the key factor in promoting cancer progression in LUAD patients. Whether LBX2 could modulate expression of EMT‐related genes, such as E‐cadherin, N‐cadherin, and vimentin was then tested. The western blotting assays indicated that E‐cadherin was significantly upregulated in LBX2‐knockdown cells and it was remarkably downregulated in LBX2‐overexpressed cells. N‐cadherin and vimentin were significantly downregulated in LBX2‐knockdown cells and were remarkably upregulated in LBX2‐overexpressed cells (Fig 6b). Overall, our data suggest that LBX2 might participate in tumor proliferation, migration, and invasion through EMT progression.

## Discussion

LUAD has a poor prognosis due to rapid tumor progression and metastasis.^14^ In recent years, molecular targeting drugs and immune checkpoint inhibitors have been approved and widely used for advanced LUAD treatments; however, the therapeutic effects are limited to short intervals of time.^6^, ^7^, ^15^ As the mechanism of cancer development is still relatively unknown, it is crucial to identify the genetic alterations underlying key steps in LUAD formation and progression and seek for new biomarkers and therapeutic targets for this type of cancer.

By analyzing information from TCGA database, LBX2 was identified as a LUAD‐related gene that is significantly upregulated in LUAD tissues. Moreover, it was seen that high expression levels correlated with a poor prognosis. It is also known that other homeobox genes, the ladybird homeobox gene homologues (LBX), play an important role in body pattern formation and morphogenesis in vertebrate animals. A recent study showed that LBX2 is expressed in various types of cancers and might be involved in tumor development.^16^, ^17^, ^18^ In this study, we determined that LBX2 might also have an important function in LUAD development. We observed that LBX2 was abnormally expressed in LUAD tissues and that expression levels correlated with patient clinicopathological characteristics and OS. In order to explore the function of LBX2 in LUAD, we chose several LUAD cell lines to perform experiments in vitro. As expected, we found that LBX2 was highly expressed in LUAD cells. We also found that overexpressing or knocking down the LBX2 gene could increase or inhibit cell proliferation, migration, and invasion capacities, respectively. in vivo experiments showed that LBX2 overexpression increased tumor growth and metastasis.

To explore the possible underlying mechanism of action of LBX2 in LUAD, microarray data were retrieved from the GEO database. By analyzing these data, we found that LBX2 participated in the EMT progression of LUAD and determined that EMT was an important step in LUAD invasion and metastasis. To verify this, we measured the protein levels of the EMT‐related genes, namely, E‐cadherin, N‐cadherin, and vimentin in LBX2‐knockdown cells and LBX2‐overexpressed cells. Results showed that LBX2 could regulate the expression of EMT‐related genes in LUAD. These regulations might be the reason for the promotion of LUAD progression by LBX2. These findings deserve further exploration to elucidate the mechanism underlying this regulation.

In summary, our study suggests that LBX2 may regulate EMT progression, leading to increased cell proliferation, migration, and invasion of LUAD. Our findings indicate that LBX2 might be both a potential biomarker and therapeutic target for LUAD.

## Disclosure

No authors report any conflict of interest.
